# Mr. Dawson, on Procidentia Uteri

**Published:** 1805-02-01

**Authors:** G. P. Dawson

**Affiliations:** Member of the Royal College of Surgeons in London. Sunderland


### *To the Editors of the Medical and Physical Journal.*

GENTLEMEN,

FEW complaints are, I believe, more distressing to females than Procidentia Uteri; but how much more distressing must it be when accompanied with a laceration of the perinæum, I leave any one to judge. It is a complaint which, in a great measure, renders the unhappy sufferer unfit for society; and, surely, any thing which tends to lessen the inconvenience she labours under, and make her life more comfortable, must be a matter of some, if not of great importance. Such is the subject of the case I am about to relate, and which I take the liberty of requesting you to insert in your valuable publication. Twenty years ago, a lady, in her first and only child, had unfortunately her perinæum lacerated by a speedy labour. Her misfortune first became known to her by that worst of all symptoms, viz. a want of retentive power over the *fæces*; but how much more was her distress aggravated when she discovered a small tumour projecting as far as the *labia pudendi*. From motives of female delicacy, not, alas! uncommon, though certainly blameable, this lady kept her unfortunate complaint a profound secret for fourteen years, without having recourse to any means whatever. At the expiration of this time, which was in the winter of 1798, she became more alarmed than usual, in consequence of a sudden protrusion of the tumour a considerable way beyond the external labia; and now she became afflicted with a compleat procidentia uteri. From peculiar circumstances I at this time became her confidant; she was now in a very unpleasant situation, the tumour was almost continually down when she was not in a horizontal position, and was attended with a sharp and whitish discharge. Though perhaps not at this time very intimately acquainted with the true nature of this lady's complaint, I was yet very well convinced that she could not wear any of those kind of pessaries which are recommended by writers for procidentia uteri attended with a lacerated perinæum. Those who are partial to such pessaries may possibly ask, how was I convinced the lady had never a pessary of any kind? how then could I determine? To this I would answer, that the vagina was in so irritable a state as hardly to bear the touch of a finger; and when I mentioned the wearing of a pessary, she absolutely shuddered at the idea. I therefore merely advised her to wear a common T bandage. This bandage was of some, though not of great use; it had the effect of preventing a protrusion beyond the external parts, but this was all, for the tumour still came down as far as the *labia pudendi,* accompanied with a considerable and unpleasant discharge, which by its excoriating, was of course productive of much trouble and distress. While I was in London in the winter of 1803, the recollection of this lady's case induced me to pay a more than ordinary attention to this subject, while prosecuting my studies at St. Thomas's and Guy's Hospitals. Dr. Haighton, who lectures on midwifery at Guy's, was of opinion that the different pessaries contrived for procidentia uteri were but of little service, but strongly recommended the use of a sponge one. Fully conscious of the value of this eminent teacher's advice, I secretly resolved to make trial of it, in the case I am relating, the first opportunity. In June, 1804, I had the pleasure of seeing this lady, and found she was still continuing the use of the T bandage. Being in a very uncomfortable situation, and unable to walk above a mile without experiencing great inconvenience, I now directed the bandage to be discarded, and requested her to wear a pessary which I would prepare. I will confess I was rather doubtful of its success, for though I had heard Dr. Haighton recommend it, yet I was fearful that it might not have the good effect while that procidentia uteri was likewise accompanied with a lacerated perinæum. The trial was however made, and it succeeded beyond my most sanguine wishes. The manner in which I made the pessary was extremely simple. I took a roundish piece of common sponge, and sewed to its middle the end of a piece of tape several inches long. I now desired this sponge to be introduced up the vagina as far as possible, and then to be allowed to remain. This done, a few inches of the tape hung externally, which was pinned to her linen. The tape attached in these two ways answered two good purposes; first, it prevented the sponge falling to the ground in case of its escape from the vagina; secondly, it afforded an opportunity of drawing it out at any time when necessary. The lady now found herself compleatly relieved, and she, who for many years had been in much distress, and unable to walk to any great distance, was now by a very simple contrivance extremely comfortable, and, to use her own expression "as well able to walk to any distance as she had ever been in her juvenile days." I therefore recommended two of these pessaries to be made; a clean one one to be introduced every morning, and the other to be immersed in any simple antiseptic water. It has, I believe, with some, been customary to make pessaries of sponge in a different manner from the plan I have adopted, and that is by forming a piece of sponge into a round ball, and then confining it in that form by strongly tying it with twine twisted in a variety of different ways. To this mode of making a pessary I would object; prepared in this manner it is merely a firm and compact body; the advantages of the sponge are, I may say, lost; and I think it is but fair to conclude that a pessary, made in such a manner, would irritate equally as much as a pessary made of any other substance. For the advantages of a sponge pessary are, its occasioning no pain, and its capability of expansion upon the application of moisture. If these are its acknowledged advantages, I will then say, that neither of them can be derived from the pessary made in the manner I have last stated. It cannot expand, for it is firmly bound down with twine at every part! It cannot be easy to the patient, for the friction of the cord against the vagina must produce pain! May not this mode of preparation have been the reason why, in some places, the sponge pessary has not been brought into more general use? If then it has proved of such utility in the instance I have recited, surely its effect will be doubly useful in simple cases of prolapsus and procidentia uteri. From the observations I have been able to make myself, and from the still more important ones of others, I am inclined to think there is no doubt of the manifest superiority which the sponge pessary holds over every other. If, indeed, we reflect but for a moment, how should it be otherwise? The other kind of pessaries are made of box wood, or ivory; these substances are hard and irritating, difficult to introduce, and when introduced, so painful as hardly to be borne. Independent of these, they have other disadvantsges, for the discharge which we always have in procidentia uteri from irritation, is either confined by these pessaries, or else allowed to pass out by means of an aperture, either of which must necessarily be inconvenient and unpleasant. Not so is it with the sponge pessary. It is soft, pliable, and easy of introduction, occasions no pain, and from its known absorbent quality, readily takes up all the discharge and moisture. I have not, gentlemen, made these remarks from vanity, or requested their publication under an idea that I derive any credit from them, for surely it were madness to think I could; any person, under similar circumstances, might have

 done done the same, nay perhaps better. My only object was to write a true and simple case, in hopes of proving that the sponge pessary has decidedly the advantage over every other; to shew, that in a case of procidentia uteri with a lacerated perinæum, it succeeded most effectually; and to hope it will be the means of inducing practitioners to have recourse to it in cases of a like nature.

I am, &c.

*Sunderland, Dec.* 10, 1804. G. P. DAWSON,

Member of the Royal College of Surgeons in London.

